# Fetal Macrophages Exposed to *Salmonella* Antigens Elicit Protective Immunity Against Overwhelming *Salmonella* Challenge in A Murine Model

**DOI:** 10.3390/biomedicines9030245

**Published:** 2021-03-01

**Authors:** Jeng-Chang Chen, Liang-Shiou Ou, Ming-Ling Kuo, Li-Yun Tseng, Hsueh-Ling Chang, Shiang-Chi Chen, Cheng-Hsun Chiu

**Affiliations:** 1 Department of Surgery, Chang Gung Children’s Hospital, College of Medicine, Chang Gung University, Taoyuan 333, Taiwan; 2 Division of Allergy, Asthma and Rheumatology, Department of Pediatrics, Chang Gung Children’s Hospital, College of Medicine, Chang Gung University, Taoyuan 333, Taiwan; ouliangshiou@gmail.com (L.-S.O.); mingling@mail.cgu.edu.tw (M.-L.K.); 3 Department of Microbiology and Immunology, Graduate Institute of Biomedical Sciences, College of Medicine, Chang Gung University, Taoyuan 333, Taiwan; 4 Pediatric Research Center, Chang Gung Children’s Hospital, College of Medicine, Chang Gung University, Taoyuan 333, Taiwan; babbi0519@hotmail.com (L.-Y.T.); shiee@cgmh.org.tw (H.-L.C.); 5 Department of Nursing, Taipei Medical University, Taipei 110, Taiwan; rossie7766@gmail.com; 6 Division of Pediatric Infectious Diseases, Department of Pediatrics, Chang Gung Children’s Hospital, College of Medicine, Chang Gung University, Taoyuan 333, Taiwan; 7 Molecular Infectious Disease Research Center, Chang Gung Memorial Hospital, Taoyuan 333, Taiwan

**Keywords:** macrophage, fetal immunization, *Salmonella*, maternal infection, vertical transmission

## Abstract

Despite the evidence for fetal immunization following maternal infection, it remained a mystery how the fetal immune system was primed by vertically-transmitted pathogens or microbial antigens, especially before its full maturation. We previously demonstrated the capacity of fetal macrophages for endocytosing oncoprotein and allergens to bridge towards adaptive immunity in postnatal life. To investigate the immunological consequences of fetal contact with microbial antigens and the role of fetal macrophages in the defense against infection before T-cell development, we exposed gestational day 14 murine fetuses and their macrophages to flagellin and heat-killed *Salmonella* Typhimurium. Recipients with in utero exposure to *Salmonella* antigens or adoptive transfer of microbial antigen-loaded fetal macrophages were examined for immune responses to *Salmonella* antigens and resistance to virulent *Salmonella* challenge. Fetal exposure to microbial antigens or adoptive transfer of microbial antigen-loaded fetal macrophages could confer antigen-specific adaptive immunity. However, protective immunity against lethal *Salmonella* challenge was only granted to those receiving heat-killed *Salmonella* antigens, presenting as heightened recall responses of serum anti-lipopolysaccharide immunoglobulins and interferon-gamma. In immunized recipients surviving *Salmonella* challenge, their serum transfer to succeeding recipients provided immediate protection from lethal *Salmonella* challenge in preference to lymphocyte transfer, indicating a more active role of humoral immunity in the prevention of *Salmonella* invasiveness. Our study sheds insight on the role of fetal macrophages in immunogenicity to transplacental pathogens regardless of fetal lymphocyte maturity, paving the way for fetal macrophage therapies to enhance vaccine responsiveness or increase resistance to pathogenic microorganisms in perinatal life.

## 1. Introduction

Maternal infections during pregnancy may be accompanied by vertical transmission of pathogens such as protozoa, bacteria, and viruses to the fetuses [1,2]. Under such circumstances, the fetal immune system must take an action to deal with invading pathogens or pathogen-associated antigens. The development of pathogen-specific B- or T-cell responses in the newborns exposed in utero to maternal infection indicates an event of in utero priming [3,4], whereas an increased susceptibility to homologous pathogens in postnatal life represents in utero induction of tolerance [3,5]. Since Medawar’s discovery of actively acquired tolerance to allogeneic antigens [6], there has been a general acceptance that T-cell maturity determined whether fetal antigen exposure would be tolerogenic or immunogenic. In humans, single positive CD4^+^ or CD8^+^ thymocytes show up in the thymus, and emigrate in the fetal liver and spleen by the beginning of the second trimester [7]. Thus, the human T-cell system in the first trimester of gestation is regarded as immature, having a tendency towards tolerization. However, it remains obscure whether maternal infection in the first trimester is apt to induce fetal tolerance to microbial antigens. Notably, the tolerant phenotype of offspring was a rarity in the literature, mainly confined to maternal filariasis [8] or malaria [9], and closely related to peripheral regulatory mechanisms [3,5].

Over the past few decades, there has been a large body of evidence showing that in utero exposure to alloantigens before full T-cell maturation did not consistently induce allogeneic graft tolerance [10,11,12]. Some animal studies even yielded conflicting results of alloreactivity to transplantation antigens in developing fetuses [13,14,15]. In our murine studies, effector functions by all arms of an adaptive immune system developed following in utero exposure to soluble peptide antigens regardless of lymphocyte maturity [16,17]. Such an event of in utero immunization could be attributed to fetal macrophage-like phagocytes (FMs), capable of taking up antigens for downstream immune signaling of antigen presentation [16]. In humans, maternal infection was reported to evoke innate immune responses in the fetus [3]. As a result, innate FMs probably take part in the control of fetal immune responses to vertically transmitted pathogens or their antigens, but remain poorly characterized. To investigate the immunological consequences of prenatal exposure to microbial antigens and the role of FMs in the defense against infections, we exposed pre-immune murine fetuses and their FMs to antigens from nontyphoid *Salmonella*, which was a model microorganism and could vertically spread to fetuses in human maternal infection [18,19]. We showed that FMs played a role in the in utero induction of adaptive immune responses to *Salmonella* antigens. When loaded with heat-killed *Salmonella*, FMs could elicit humoral immunity with heightened Th1-skewed cytokine in the recipients, sufficient to protect against lethal *Salmonella* challenge.

## 2. Materials and Methods

### 2.1. Mouse Husbandry

Inbred FVB/N mice were purchased from the National Laboratory Animal Center (Taipei, Taiwan) at the age of 6–8 weeks, and housed in the animal care facility at Chang Gung Memorial Hospital (CGMH) under the standard guidelines from “Guide for the Care and Use of Laboratory Animals” and with the approval of the CGMH Committee on Animal Research (IACUC2014092202; 22/09/14). Females were caged with males in the afternoon and checked for vaginal plugs the following morning. The day of the plug being observed was called day 0 of the pregnancy.

### 2.2. In Utero Injection [20,21]

Under anesthesia, the uteri of gestational day 14 pregnant mice were exposed through a vertical laparotomy. A 60 µm glass micropipette with beveled tip was used to inject endotoxin-free recombinant flagellin (RecFLA-ST 0.1–0.3 μg, from *Salmonella (S.)* Typhimurium, InvivoGen, San Diego, CA, USA) or heat-killed *S.* Typhimurium SL1344 (97–100 °C for 30 min in thermomixer) in 5–10 µL saline into the peritoneal cavities of all fetuses in a litter via trans-uterine approach. Then the abdomen was closed in two layers of 5–0 silk suture. After the operation, all mice were housed in an undisturbed room. Pups were weaned at the age of 3 weeks.

### 2.3. Harvest of Gestational Day 14 FMs from the Liver and Peritoneum [17]

Under anesthesia for pregnant mice, midline laparotomy was performed to expose the uteri. The fetus was delivered through hysterotomy and immediately washed with saline. Then fetal peritoneal cells were collected by peritoneal lavage with 0.5 mL saline. Subsequently, the liver was obtained and dissociated by passage through 70 µm cell strainers (Becton Dickinson, Franklin Lakes, NJ, USA) to obtain fetal leukocytes. FMs were then enriched by Ficoll density gradient centrifugation at 600× *g* for 25 min. Enriched FMs were washed with PBS (phosphate buffered saline) for further use.

### 2.4. Adoptive Transfer of Flagellin or Bacteria-Loaded FMs

Pooled cells from the fetal peritoneal cavity and dissociated livers were subjected to Ficoll density gradient centrifugation to enrich the FMs. The enriched FMs (≥80%) were then treated with flagellin (2 × 10^7^ cells/1 μg flagellin) or heat-killed *S.* Typhimurium in Roswell Park Memorial Institute (RPMI) media overnight, and washed vigorously with PBS. Then they were intravenously injected into adult mice (8–12 weeks old) at a dose of 2 × 10^7^ cells/mouse in 200 μL saline. Within 1–2 months after cell transfer, recipients were examined for the production of antigen-specific immunoglobulin G_1_ (IgG_1_) and their resistance to intraperitoneal challenge of live *S.* Typhimurium. Controls were FMs maintained in flagellin or bacteria-free medium.

### 2.5. Intraperitoneal Challenge of S. typhimurium in Mice

Enumeration by colony forming unit (CFU) provided a direct measurement of bacterial cell counts. Quantification of *S.* Typhimurium SL1344 in batch culture was determined using CFUs as estimated by plating dilutions and optical density measurements to generate a standard curve of CFU vs. optical density [22]. The susceptibility to *S.* Typhimurium infection in FVB/N mice was evaluated by intraperitoneal challenge of live *S.* Typhimurium (10^1^–10^7^ bacteria/mouse). Then the mice were monitored daily for 2 weeks or until the day of death. The survival curve was constructed with the Kaplan–Meier method.

### 2.6. Electron Microscopic Examination for Endocytosis of FMs

Pooled FMs were pulsed with either gold nanoparticles or pathogens of *S.* Typhimurium or *Staphylococcus aureus* overnight, and washed vigorously with PBS. Cell pellets were fixed with 4% formaldehyde and 1% glutaraldehyde in 0.1 M phosphate buffer (21.8 g Na_2_HPO_4_ + 6.4 g NaH_2_PO_4_ + 1 L distilled water, pH = 7.4) and then subjected to sequential dehydration procedures (ethanol 50% × 15 min, 70% × 15 min, 95% × 15 min, and 100% × 15 min twice), followed by 100% propylene oxide 2 × 15 min; EMBed 812 (Resin, Electron Microscopy Sciences, Hatfield, PA, USA): propylene oxide (1:1) for 1–2 h and EMBed 812:propylene oxide (2:1) overnight in a desiccator with the top off. Then the samples were embedded in beam capsules and baked in a 60 °C oven for 48 h. Ultra-thin sections were taken and placed on grids. The grids were sent for staining with uranyl acetate for 15 min, rinse with distilled water, and then stained with lead citrate for 3–5 min. The FMs were examined for endocytosis under transmission electron microscopy.

### 2.7. ELISA for Serum Anti-Flagellin and Anti-LPS IgG_1_ Levels

Four to six weeks after injection, the murine recipients were subjected to blood sampling to measure serum anti-LPS (lipopolysaccharide) or anti-flagellin IgG_1_ levels. ELISA (enzyme-linked immunosorbent assay) microtiter plates (Corning, Corning, NY, USA) were coated with 50 μg/mL LPS (from *S.* Typhimurium, Sigma-Aldrich, St. Louis, MO, USA) or 400 ng/mL flagellin (from *S.* Typhimurium) [23], blocked with 3% bovine serum albumin (BSA, Sigma-Aldrich) in PBS, and incubated with 100 μL of diluted samples. In each well, biotinylated anti-mouse IgG_1_ (Clone RMG1-1, BioLegend, San Diego, CA, USA) was added and followed by streptavidin-horseradish peroxidase (Sigma-Aldrich). The reaction was developed by adding 100 µl NeA-blue tetramethylbenzidine substrate (Clinical Science Products, Mansfield, MA, USA) and stopped with 2 M H_2_SO_4_. The optical density at 450 nm was read using an ELISA reader. Serum anti-LPS and anti-flagellin IgG_1_ levels were determined by the standard curve of mouse anti-*S.* Typhimurium LPS IgG_1_ mAb (monoclonal antibody 1E6, Abcam, Cambridge, UK) and mouse anti-flagellin IgG_1_ mAb (Clone FLIC-1, BioLegend).

### 2.8. Proliferation of Lymphocytes in Response to Flagellin

Splenic lymphocytes were enriched by density gradient centrifugation and then cultured in triplicate each with 2 × 10^5^ cells in 200 μL RPMI 1640 medium containing 10% fetal calf serum in 96-well plates. Responder lymphocytes were stimulated by flagellin (0.5 μg/mL), BSA (0.5 μg/mL), or Con-A (1 μg/mL). For the measurement of lymphocyte proliferation, day 5 cells were first subjected to 16 h incubation with tritiated thymidine (ICN Biomedicals, Erie, PA, USA) at a final concentration of 1 μCi per well and then harvested to count the incorporated tritium in a liquid scintillation counter (1450 Microbeta Plus counter, Wallac, Helsinki, Finland). Lymphocyte proliferation was determined by the readout of incorporated tritium as counts per minute.

### 2.9. Quantification of IFNγ, IL4, and IL5 Cytokines

Enriched splenic lymphocytes were cultured in triplicate at a density of 5 × 10^6^ cells/mL in 500 μL RPMI 1640 with 10% fetal calf serum under the stimulation of flagellin (0.5 μg/mL) for 5 days. Then supernatants of cell cultures were collected. Blood was sampled from murine recipients for serum collection before and one week after adoptive transfer of FMs with or without a load of heat-killed *Salmonella*, and one week after intraperitoneal *Salmonella* challenge as well. Cytokines of IFNγ, IL4, or IL5 in culture supernatants and sera were quantified using ELISA assay kits according to the manufacturer’s protocols (BioLegend).

### 2.10. Statistical Analyses

All bar data are shown as 95% confidence intervals for the means. The equality of means was examined by Student’s *t*-test between two independent or paired groups, or by one-way analysis of variance (ANOVA) among three or more groups with post hoc Fisher’s least significant difference (LSD) multiple comparisons. Plots of survival time were constructed with the Kaplan–Meier method and compared by the log-rank test. Differences were regarded as significant in all tests at *p* < 0.05.

## 3. Results

### 3.1. In Utero Injection of Flagellin in FVB/N Mice

**Figure 1:**
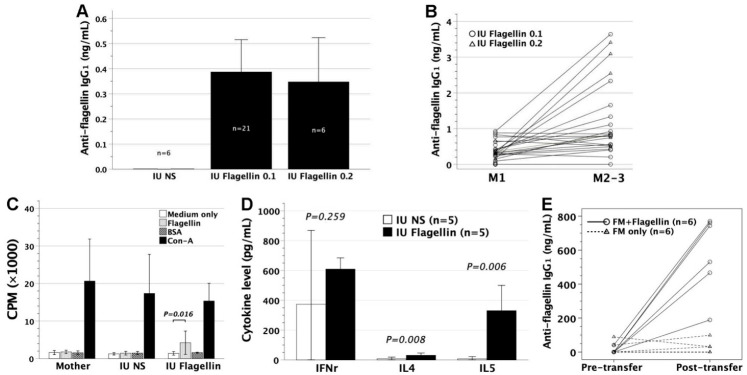
Immunological consequences after in utero injection of flagellin and adoptive transfer of flagellin-laden FMs. (**A**) FVB/N mice were subjected to in utero injection of 0.1 or 0.2 μg endotoxin-free flagellin (IU Flagellin 0.1 and 0.2) at gestational day 14. At the age of about 1 month, mice were examined for serum anti-flagellin IgG_1_ levels by ELISA using mouse monoclonal IgG_1_ antibody (FLIC-1, BioLegend) as the standard curve. The controls were sera from mice with in utero injection of saline (IU NS). The IU Flagellin 0.1 and 0.2 groups significantly secreted anti-flagellin IgG_1_ as opposed to the IU NS group (*p* = 0.001 and 0.017, respectively). There was no difference in anti-flagellin IgG_1_ levels between flagellin doses of 0.1 and 0.2 μg (*p =* 0.724). (**B**) Anti-flagellin IgG_1_, rechecked at the age of 2–3 months (M2–3), rose to a significantly higher level (*p =* 0.001, paired *t*-test) than that at 1 month old (M1). (**C**) The splenic lymphocytes of recipients (n = 3 for each of IU Flagellin and IU NS) and mothers (n = 3) were examined for their proliferative responses to flagellin in culture systems by the readout of incorporated tritium as CPM. Medium only was used as a background control, BSA as a third-party stimulator, and Con-A as a mitogen to stimulate T-cell population. IU Flagellin exhibited proliferative responses specifically to flagellin stimulation (LSD following ANOVA), whereas IU NS and the mother were unresponsive. (**D**) Lymphocyte polarization was examined by IFNγ, IL4, and IL5 secretions in cell cultures under flagellin stimulation. IU Flagellin compared favorably in the levels of IL4 and IL5 but not IFNγ to IU NS, indicating a Th2-skewed phenotype. (**E**) Enriched FVB/N FMs were pulsed with flagellin overnight, and then intravenously injected into adult FVB/N mice (FM + Flagellin). Serum anti-flagellin IgG_1_ levels before and after the cell transfer (4–6 weeks later) were measured by ELISA as described before. FM + Flagellin elicited significantly higher anti-flagellin IgG_1_ (*p* = 0.002, paired *t*-test) than the transfer of FMs maintained in media overnight (FM only, *p* = 0.752). Coupled circles or triangles represent paired pre- and post-transfer data from an individual mouse. FM, fetal macrophage; CPM, counts per minute; Con-A, concanavalin A; LSD, least significant difference

To examine the immune responses of murine fetuses to microbial antigens, we intraperitoneally injected 0.1, 0.2, or 0.3 μg flagellin (from *S.* Typhimurium) into gestational day 14 FVB/N murine fetuses. The fetal recipients failed to survive 0.3 μg flagellin. At the age of 1 month, mice were examined for the levels of serum anti-flagellin IgG_1_ by ELISA (Figure 1A). In utero injection of 0.1 or 0.2 μg flagellin caused the generation of anti-flagellin IgG_1_. Serum anti-flagellin IgG_1_ levels did not differ between the injected doses of 0.1 and 0.2 μg. At the age of 2–3 months, anti-flagellin IgG_1_ levels persisted and even significantly increased compared with those at 1 month old (Figure 1B), arguing against transplacental IgG_1_ of maternal origin. The lymphocytes of flagellin recipients were examined for in vitro proliferation by tritium incorporation in postnatal life. They significantly proliferated specifically in response to flagellin stimulation, as opposed to their saline controls (Figure 1C). Notably, maternal lymphocytes were unresponsive to flagellin, indicating the absence of inadvertent maternal sensitization by flagellin in the process of in utero flagellin injection. Assessed by IFNγ, IL4, and IL5 secretion under flagellin stimulation in cell culture systems, the lymphocytes of flagellin recipients were polarized towards Th2-skewed phenotypes (Figure 1D).

### 3.2. Adoptive Transfer of Gestational Day 14 FMs Pulsed with Flagellin into FVB/N Recipients

Pooled FMs were first pulsed with flagellin overnight. To evaluate the ability of flagellin-loaded FMs to present flagellin antigens, we intravenously transferred the flagellin-loaded FMs into 1–2-month-old FVB/N mice. Four to six weeks after cell transfer, the recipient mice were examined for their serum anti-flagellin IgG_1_ levels. Flagellin-loaded FMs caused the generation of anti-flagellin IgG_1_ in sera, as opposed to FMs without flagellin loading (Figure 1E). This indicated the development of adaptive immune responses, as observed in the fetuses exposed to flagellin in utero.

### 3.3. Susceptibility of FVB/N Mice to S. typhimurium Infection

**Figure 2:**
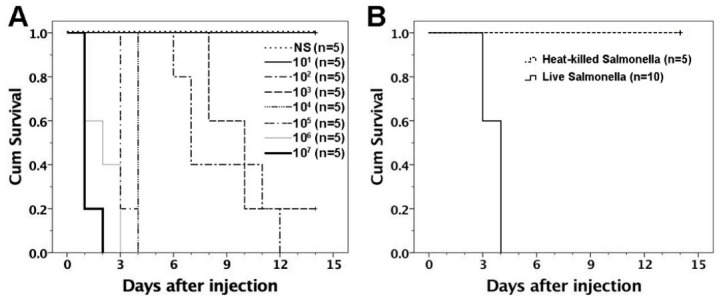
Survival curves after intraperitoneal injection of *S.* Typhimurium in FVB/N mice. (**A**) FVB/N mice at 2–3 months old were subjected to intraperitoneal injection of *S.* Typhimurium at various doses of 10^1^–10^7^. Mice died of infection when the *Salmonella* dose was ≥ 10^2^. The survival of mice following intraperitoneal *Salmonella* challenge was dose responsive (NS-10^1^ vs. 10^2^–10^3^, *p =* 0.002–0.014; 10^2^–10^3^ vs. 10^4^–10^5^, *p* = 0.002–0.003; 10^4^–10^5^ vs. 10^6^–10^7^, *p* = 0.002–0.042). (**B**) Live and heat-killed *S*. Typhimurium of 10^5^ were intraperitoneally injected into 2–3-month-old FVB/N adults. Live bacteria killed the hosts within 3–4 days after injection but heat-killed bacteria were not lethal to the hosts after 2 weeks (*p* = 0.001). NS, normal saline

We examined the susceptibility of adult FVB/N mice (2–3 months old) to *S.* Typhimurium infection. FVB/N adults were subjected to intraperitoneal injection of *S.* Typhimurium at doses of 10^1^–10^7^ per mouse. The survival of mice was then monitored for two weeks. With the *Salmonella* dose of ≥ 10^2^, mice presented specific behaviors such as reduced activity and feeding, ruffled fur, hunched positions, ataxia, and tremors. Mice died of *Salmonella* infection within one to two weeks after the *Salmonella* dose of 10^2^–10^3^, within three to seven days after the dose of 10^4^–10^5^, and within three days after the dose of 10^6^–10^7^ (Figure 2A). To further test the influence of bacterial viability on its toxicity to the hosts, we injected live and heat-killed *S.* Typhimurium of 10^5^ intraperitoneally into FVB/N adults. It showed that only live bacteria were lethal to the hosts (Figure 2B), suggesting that the bacterial viability contributed to its lethal effects.

### 3.4. Intraperitoneal Challenge of S. typhimurium in Murine Recipients

**Figure 3:**
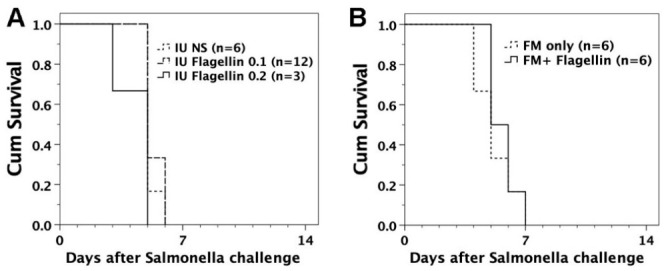
Intraperitoneal *S.* Typhimurium challenge. Six to eight weeks after the treatment of in utero flagellin injection or adoptive transfer of flagellin-loaded FMs, the recipient mice were subjected to intraperitoneal challenge of 10^5^ live *S.* Typhimurium SL1344. (**A**) Mice with in utero flagellin injection (IU Flagellin 0.1 and 0.2) did not differ in survival (*p* = 0.470 and 0.152) from those with saline injection (IU NS). (**B**) There was no significant difference in survival (*p* = 0.511) between adult recipients with injection of flagellin-loaded FMs (FM + Flagellin) and FMs without flagellin loading (FM only).

FVB/N mice with in utero flagellin injection or adoptive transfer of flagellin-loaded FMs were examined for the ability to defend against lethal challenge of *S.* Typhimurium. They were subjected to intraperitoneal injection of live *S.* Typhimurium (10^5^ bacteria/mouse). Neither in utero injection of flagellin nor adoptive transfer of flagellin-loaded FMs could protect the recipients from lethal challenge of live *S*. Typhimurium (Figure 3).

### 3.5. Endocytosis of Pathogens by FMs

**Figure 4:**
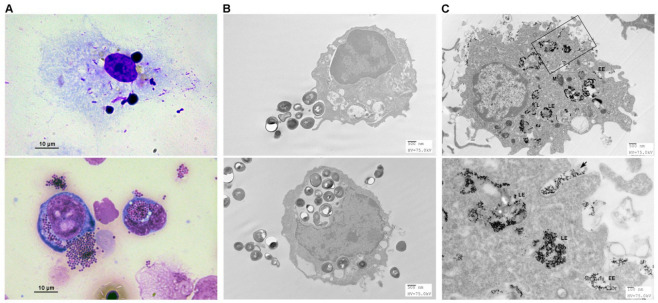
Endocytosis of pathogens or gold nanoparticles by FMs. FMs were grown on coverslips and fed with pathogens (*S.* Typhimurium or *Staphylococcus* aureus) or 5 nm gold particles for 12 h. Then they were subjected to Diff-Quik staining or sample preparation for transmission electron microscopy. (**A**) Diff-Quik staining revealed the capacity of FMs for pathogen phagocytosis. *Salmonella* were taken up in solitude by FMs (upper panel), whereas Staphylococci were engulfed in grape-like clusters (lower panel). (**B**) In electron microscopic examinations, FMs stretched their pseudopodia to engulf the large particles of 0.5–1 µm bacteria. This phagocytosis led to a huge phagosome by the fusion of the cell membrane. (**C**) FMs took up gold nanoparticles by invagination (arrow, lower panel) of the cell membrane to form a vesicle as the endosome. The early endosomes (EE) had a uniform, electronlucent appearance without much internal material. The late endosomes (LE) contained closed-packed luminal vesicles with internal membrane fragments. The lysosomes (L) appeared to be more homogeneous in structure near the nuclei. The mitochondria (M) are easily recognized by their internal cristae.

Pooled FMs (5 × 10^6^ cells/mL RPMI) were treated in vitro with *S.* Typhimurium (bacilli), *Staphylococcus aureus* (cocci) bioparticles, or 5 nm gold particles for 12 h. Following staining or processing, they were subjected to light or transmission electron microscopic examinations. FMs were able to engulf bacilli (1–2 µm) or cocci (0.5–1 µm) (Figure 4A). On electron microscopic examinations, large bacterium bioparticles were endocytosed by pseudopodia to form a huge phagosome (Figure 4B), but small gold nanoparticles suspended in extracellular fluid were brought into the cells through an invagination of the cell membrane (Figure 4C). The whole endocytic pathways within FMs could be visualized by gold nanoparticle labelling in electron microscopic examinations [24]. The capacity of FMs to endocytose pathogens indicated their ability to take part in the defense against infection.

### 3.6. In Utero Injection of Heat-Killed S. typhimurium into FVB/N Murine Fetuses

**Figure 5:**
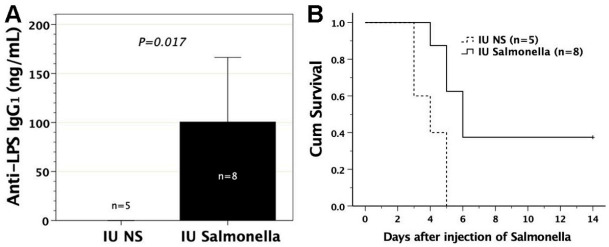
Survival of recipients with in utero exposure to heat-killed *Salmonella* following postnatal challenge of *S.* Typhimurium. Gestational day 14 FVB/N fetuses were subjected to intraperitoneal injection of 5 × 10^3^ heat-killed *S.* Typhimurium SL1344 (IU *Salmonella*). (**A**) Serum anti-LPS IgG_1_ levels were examined at the age of 1 month by ELISA using mouse monoclonal anti-LPS IgG_1_ (1E6, Abcam) as the standard curve. Recipients with IU *Salmonella* significantly generated anti-LPS IgG_1_. (**B**) At the postnatal age of 6–8 weeks, the recipients were challenged intraperitoneally with 10^5^ live *S*. Typhimurium. IU *Salmonella* compared favorably in survival to mice receiving in utero saline injection (IU NS, *p* = 0.011). LPS, lipopolysaccharide.

To test whether in utero exposure to *Salmonella* antigens could elicit immunity against *Salmonella*, we subjected gestational day 14 FVB/N murine fetuses to the intraperitoneal injection of heat-killed *S.* Typhimurium, beginning at a dose of 10^8^. The murine fetuses could not survive the injection of heat-killed *Salmonella* until a dose of 5 × 10^3^ heat-killed *Salmonella* was used. This might be due to endotoxin release from the killed *Salmonella,* which was detrimental to the pregnancy. Eight recipient mice survived prenatal exposure to 5 × 10^3^ heat-killed *S.* Typhimurium and significantly generated anti-LPS IgG_1_ in sera (Figure 5A). Among them, three showed resistance to postnatal intraperitoneal challenge of 10^5^ live *S.* Typhimurium but the five others died of *Salmonella* infection within 4 to 6 days after challenge, as compared with the survival of three to five days in the saline controls. Overall, the recipients with in utero exposure to heat-killed *Salmonella* experienced superior survival to their saline controls following postnatal *Salmonella* challenge (Figure 5B). In the three recipients surviving *Salmonella* infection, their serum IFNγ increased from 14.2, 90.2, and 108 pg/mL before challenge to 1356, 568, and 190 pg/mL after challenge, respectively, whereas serum IL4 was not detectable before or after challenge.

### 3.7. Adoptive Transfer of FMs Loaded with Heat-Killed S. typhimurium

**Figure 6:**
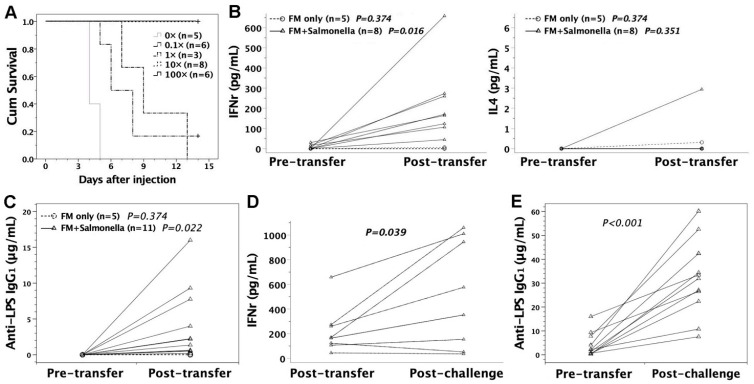
Immunological outcome following adoptive transfer of FMs loaded with heat-killed S. Typhimurium. FVB/N adults were intravenously injected with 2 × 10^7^ FMs loaded with heat-killed *S.* Typhimurium SL1344 (0×, 0.1×, 1×, 10×, and 100× in amount) overnight. (**A**) Four weeks later, the recipients were tested for their resistance to lethal challenge of 10^5^ live *S.* Typhimurium. With heat-killed *Salmonella* of 0.1× and 1×, the recipients experienced better survival (*p* = 0.004 and 0.012, respectively) than the FM controls without *Salmonella* loaded (0×). When *Salmonella* doses increased to 10× and 100×, all recipients survived lethal challenge, achieving superior survival to *Salmonella* doses of 0.1× (*p* = 0.001 and 0.005, respectively) and 1× (*p* < 0.001 and *p* = 0.002, respectively). (**B**) Following adoptive transfer, serum IFNγ was upregulated in recipients receiving *Salmonella*-loaded FMs (FM + *Salmonella*, 10× or 100×), compared to the controls receiving FMs without *Salmonella* loaded (FM only, 0×). However, IL4 remained hardly detectable in the FM + *Salmonella* and FM-only groups. (**C**) Serum anti-LPS IgG_1_ levels were examined in FVB/N recipients following cell transfer by ELISA using mouse monoclonal anti-LPS IgG_1_ (1E6, Abcam) as the standard curve. FM + *Salmonella* significantly generated anti-LPS IgG_1_ in sera, as opposed to FM only. (**D**,**E**) In the recipients (FM + *Salmonella*) surviving *Salmonella* challenge, serum IFNγ and anti-LPS IgG_1_ were further boosted. Coupled triangles or circles represent pairwise data of pre- vs. post-transfer or post-transfer vs. post-challenge from an individual mouse.

Pooled FMs from gestational day 14 FVB/N murine livers and peritonea were pulsed with heat-killed S. Typhimurium at a *Salmonella*-to-FM ratio of 0.1–100:1 overnight. Control mice received FMs that were maintained in *Salmonella*-free media. Following vigorous washing, 2 × 10^7^ heat-killed *Salmonella*-loaded FMs were injected into six- to eight-week-old adult FVB/N mice via tail veins as cellular vaccines. One month later, the recipients were subjected to intraperitoneal challenge of 10^5^ live *S.* Typhimurium. Then the mice were monitored daily for two weeks. The recipients developed resistance to *Salmonella* infection following the adoptive transfer of heat-killed *Salmonella*-loaded FMs (Figure 6A). The complete protection from lethal *Salmonella* challenge could be achieved when the *Salmonella*-to-FM ratio was ≥ 10:1. In immunized recipients, serum cytokines of IFNγ and IL4 were examined by ELISA. Following adoptive transfer of FMs loaded with heat-killed *Salmonella* (10× or 100×), IFNγ was significantly increased as opposed to IL4 in the recipients’ sera, suggesting Th1-skewed milieu in immunized mice (Figure 6B). Antibody responses specific to lipopolysaccharide (LPS) of *S.* Typhimurium were elicited as evidenced by the generation of anti-LPS IgG_1_ in the recipients’ sera (Figure 6C), indicating the development of adaptive immune responses to *S.* Typhimurium. Of note, there were heightened recall responses of serum IFNγ and anti-LPS IgG_1_ in the recipients surviving virulent *Salmonella* challenge (Figure 6D,E).

### 3.8. Protective Effects of Sera or Lymphocytes from Mice Immunized by Salmonella-Loaded FMs

**Figure 7:**
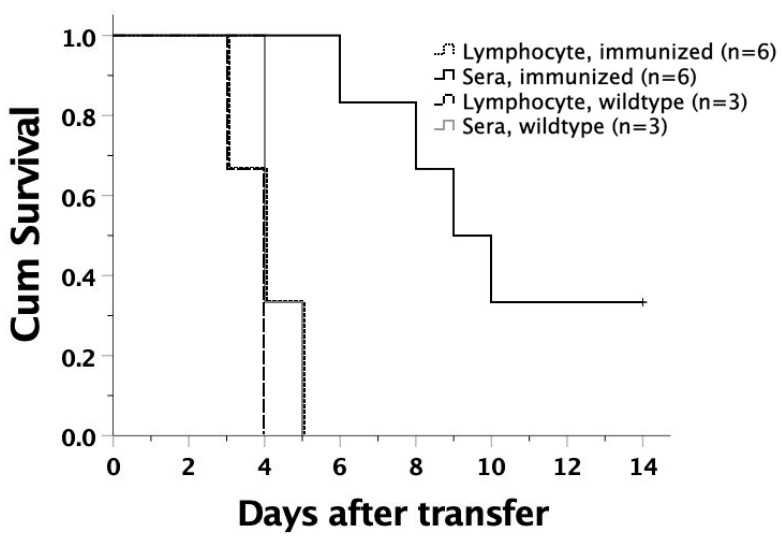
Murine survival following adoptive transfer of sera and splenic lymphocytes from the mice immunized with *Salmonella*-loaded FMs. Serum and splenic lymphocytes collected from an immunized FVB/N mouse were intravenously transferred to 6–8-week-old FVB/N adults. The recipient mice were then subjected to intraperitoneal challenge of 10^5^ live *S.* Typhimurium. Serum inocula led to better survival of the recipients than lymphocyte inocula (*p =* 0.001), and sera or lymphocytes from wildtype controls (*p* = 0.002 and *p* = 0.003, respectively). Wildtype represents FVB/N mice without any prior manipulation.

To further elucidate the mechanisms that protected the immunized mice from lethal *Salmonella* infection, we collected sera and splenic lymphocytes from the mice immunized by *Salmonella* (10×)-loaded FMs. They were intravenously transferred to six- to eight-week-old FVB/N adults. On the same day, these serum or lymphocyte recipients were then subjected to intraperitoneal challenge of virulent *Salmonella*. Serum transfer could either lengthen the survival time or prevent lethality from *Salmonella* challenge, compared to lymphocyte transfer (Figure 7). Thus, sera from immunized recipients might have immediate protection from *Salmonella* invasiveness, suggesting that humoral immunity was responsible for the immediate protection against *Salmonella* infection.

## 4. Discussion

It was reported that maternal exposure to pathogens or their related antigens might affect the offspring’s immune responses to microbial antigens [25], postnatal infection [3,26], or vaccination [27,28] even in the absence of substantial fetal infection [5]. The immunological alterations made to the offspring might result from the transplacental transfer of maternal immunoglobulins or the priming of the fetal immune system following maternal infection or vaccination [5]. Maternal antibody transmission has been a well-known phenomenon of passive immunity in fetuses, endowing maternal vaccination strategy with additional protection for the offspring in early postnatal life by boosting maternal vaccine-specific humoral immunity [29]. Independent of antibody-mediated passive immunity, in utero priming of microbial antigens was first brought to attention in 1972, as evidenced by persistent cellular immunity to the mumps virus in 10-year-old Eskimo children born to the mothers with mumps virus infection in pregnancy [25]. Subsequently, in utero sensitization as a consequence of maternal infection with mycobacteria, protozoa, helminths, and other viruses of cytomegalovirus or hepatitis B or C virus was widely reported in the literature [5]. The mechanisms of fetal sensitization to infectious antigens was attributed to the passage of pathogen-related antigens across the placenta in the form of free antigens, antigen-loaded microvesicles, or immune complexes [5,30,31]. However, much controversy still prevailed as to the capacity of the developing fetal immune system to mount adaptive immunity [5]. Transplacental antigen transmission following maternal treatment with mycobacterial antigens might contribute to recall of T-cell responses of offspring in mice [30]. However, the exact timing or quantity of antigen transmission to the murine fetus following maternal antigen exposure remained obscure. Although intrauterine inoculation of microbial antigens was not a physiological route due to bypassing the maternal interaction with antigens, free peptide antigens remained one of the transplacentally transmitted antigen formats present in the fetuses. This artificial approach enabled us to quantitatively investigate fetal immunoreactivity to microbial antigens across placental barriers at a controllable and precise gestational timing of fetal antigen exposure on gestational day 14.

It has been long known that mice immunized with attenuated *Salmonella* might mount protective immunity against secondary challenge with virulent *Salmonella* [32]. Although immune responses against *Salmonella* flagellin [32], porin [33], Vi or LPS O antigens (O polysaccharide) [34] have been reported as protective, it remains a matter of debate as to the specific antigen responsible for such protection. In this study, *Salmonella* flagellin was employed as intrauterine inocula to evaluate fetal immune responses to infectious antigens because it was a powerful immunogen [35,36], capable of inducing B- and T-cell immune responses during infection with *S.* Typhimurium in mice [32,35]. Murine T-cells were not considered functionally competent until their expression of T-cell receptors on gestational day 17 [37]. We showed that in utero exposure to soluble flagellin on gestational day 14 was not tolerogenic but rather immunogenic, as evidenced by the generation of anti-flagellin-specific immunoglobulins in postnatal life. The result directly confirmed the capacity of pre-immune fetuses to mount adaptive immunity to infectious antigens present in utero, essentially in line with the immunogenic outcomes following fetal exposure to soluble allergens [16] and oncoproteins [17]. Although passive immunization with monoclonal anti-flagellin antibodies conferred protection against lethal *Salmonella* challenge [35], active immunization by in utero exposure to flagellin was not protective.

Innate FMs developed earlier than lymphocytes as the first immune cells present during embryogenesis [38,39]. They are professional phagocytes capable of scrutinizing their surroundings so as to eliminate metabolic waste, foreign antigens, or apoptotic cells. Thus, immune protection in early gestational age to a large extent relies upon an innate phagocytic system. FMs could sequester the endocytosed antigens, differentiate toward dendritic cells, and present antigens to T-cells later in life to trigger adaptive immunity [16], leading to an event of in utero priming. Adoptive transfer of flagellin-loaded FMs consistently sensitized the recipients to generate anti-flagellin immunoglobulins. The immune responses following *Salmonella* infection in mice [40] and humans [41] have been reported as Th1-skewed phenotypes, which were a requisite for resolving infection of intracellular organisms via macrophage-activating cytokines such as IFNγ and TNFα [32,42]. However, fetal exposure to flagellin elicited Th2-skewed immune responses with enhanced IL4 and IL5 but not heightened IFNγ production, identical to Th2 immune skewness of adult mice following immunization with soluble flagellin [43,44]. This might explain why the murine recipients lacked protective immunity against virulent *Salmonella* following fetal exposure to flagellin or adoptive transfer of flagellin-loaded FMs.

Killed *Salmonella* vaccines has been used to control *Salmonella* infection and reduce mortality in animals [45], and elicit protective antibody responses in humans [46]. Injected in utero with heat-killed *S.* Typhimurium as the immunogens, murine fetuses were endowed with the ability to defend against virulent *Salmonella* in postnatal life. We showed that not only did FMs exhibit the pinocytotic capacity to ingest liquid along with the solutes (small liquid particles) by invagination of the cell membrane, but were also competent to phagocytose Gram-positive or -negative bacterial particles by stretching their pseudopodia. Following endocytosing microbial particles, their capability of evoking adaptive immunity was further examined by subjecting the mice to adoptive transfer of heat-killed *Salmonella*-loaded FMs. This macrophage-based cellular vaccine elicited immunoglobulins specific to LPS of *S.* Typhimurium and promoted IFNγ production in sera, sufficient to protect the recipients from lethality caused by intraperitoneal *Salmonella* challenge. We further showed that the passive transfer of sera rather than lymphocytes from immunized mice might increase the resistance of naive mice against lethal *Salmonella* infection. Given that vaccine-induced protection against virulent *Salmonella* demanded specific antibodies [47], the serum-transferable protection was most likely attributed to *Salmonella*-specific antibodies, which could directly bind to virulent *Salmonella* so as to impede bacterial colonization [48] and preclude their transmission [49]. Opsonization of bacteria with antibodies might also enhance phagocytosis of *Salmonella* via Fc receptor-mediated uptake to facilitate bacterial clearance [40,50]. Additionally, serum Th1 or Th1-inducing cytokines such as IFNγ or IL12 were crucial to in vivo responses against *Salmonella* infection in view of their upregulation in human *Salmonella* infection, especially the systemic salmonellosis [41]. Defective IFNγ or IL12 production or receptor signaling pathways led to heightened susceptibility to *Salmonella* infection [51,52]. In immunized hosts, IFNγ or IL12 neutralization lessened the protective immunity against *Salmonella* [53,54]. Altogether, these observations supported a critical role of Th1 cytokines in the defense against *Salmonella* infection, indicating that promoting IFNγ production in immunized mice was another serum factor relevant to the protection against *Salmonella* in this study. It is worth noting that antibodies also had an important role in amplifying the processing and presentation of *Salmonella* antigens toward CD4 T-cells to quantitatively and qualitatively affect Th1 responses [55,56]. Thus, specific antibody production and Th1 cytokine upregulation might have important implications for protective immunity in hosts immunized by cellular vaccines of *Salmonella*-loaded FMs.

## 5. Conclusions

**Figure 8:**
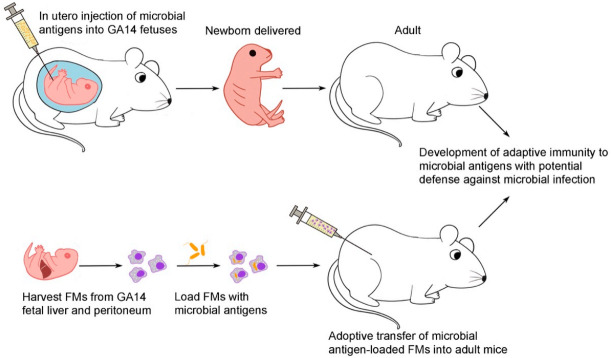
Scheme of experimental design and results. This graph summarizes the experiments and the main results to facilitate the understanding of this work. GA14, gestational day 14; FM, fetal macrophage.

Despite the clinical rarity of fetal infection with nontyphoid *Salmonella* [18,19], *S.* Typhimurium was employed in this study to investigate fetal immune responses to infectious antigens considering the high susceptibility to lethal *Salmonella* infection in mice. This proof-of-principle experiment (Figure 8) demonstrated that the developing fetuses, regardless of T-cell maturity, were competent to mount adaptive immunity in case of prenatal contact with microbial antigens and might be postnatally endowed with the capacity to defend against the pathogens from which the antigens derived. Taken together with the immunogenic outcome following in utero exposure to free peptide antigens of ovalbumin [16] and HPV E7 [17], it was hard to reconcile these results with Medawar’s actively acquired tolerance that in utero exposure to foreign antigens before full immune maturation led to tolerance. The innate fetal phagocytic system appeared to play a crucial role in triggering this acquired immune response because of its capacity for endocytosing foreign antigens at the pre-immune stage, usually referring to the period before full development of adaptive (T-cell) immunity. Thus, it may not be easy for the fetus to be “tricked” into accepting non-self antigens if innate phagocytes remain functioning well and competently. Our results cast new light on the prospects of developing novel FM cellular therapies to meet the health challenges of perinatal life, such as enhancing vaccine responsiveness or increasing resistance to pathogenic microorganisms. Perhaps we can collect cord blood for FM enrichment or expansion. Then microbial antigen-loaded FMs may be used to enhance neonatal immunity in the prevention or treatment of infectious diseases.

## Data Availability

All data are contained in this article.
